# Authors’ Reply “Comments: Evaluation of the accuracy of mammography, ultrasound, and magnetic resonance imaging in suspect breast lesions”

**DOI:** 10.6061/clinics/2021/e2980

**Published:** 2021-06-07

**Authors:** Renato de Oliveira Pereira, Larissa Almondes da Luz, Diego Cipriano Chagas, Jefferson Rodrigues Amorim, Elmo de Jesus Nery-Junior, Araci Castelo Branco Rodrigues Alves, Flávio Teixeira de Abreu-Neto, Maria da Conceicão Barros Oliveira, Danylo Rafhael Costa Silva, José Maria Soares-Júnior, Benedito Borges da Silva

**Affiliations:** I Programa de Pos-Graduacao em Ciencias da Saude, Universidade Federal do Piaui, Teresina, PI, BR; II Programa de Pos-Graduacao, Rede Nordeste de Biotecnologia (RENORBIO), Universidade Federal do Piaui, Teresina, PI, BR; III Departamento de Mastologia, Hospital Getulio Vargas, Teresina, PI, BR; IV Disciplina de Ginecologia, Departamento de Obstetricia e Ginecologia, Hospital das Clinicas HCFMUSP, Faculdade de Medicina, Universidade de Sao Paulo, Sao Paulo, SP, BR

Breast cancer is the most common cancer among the female population worldwide (1). In Brazil, a developing country, breast cancer is the most common malignancy in women after non-melanoma skin cancer (2). The success of breast cancer treatment depends on early diagnosis and treatment, which has an influence on the overall survival, regardless of the advancements in therapy (3,4). The main imaging procedures that usually lead to the early detection of breast cancer are mammography, ultrasonography, and magnetic resonance imaging (MRI). However, mammography remains the standard examination tool in the systematic screening of breast cancer (3).

Authors reply to Comments on “Evaluation of the accuracy of mammography, ultrasound, and magnetic resonance imaging in suspect breast lesions” performed by An (2020) (5). We mentioned in the Methods section that this was a retrospective study based on the review of electronic medical records from 2010 to 2018. The study aimed to compare the accuracy of the three methods (mammography, ultrasonography, and MRI) in breast lesion imaging; however, at least one of these methods would have to show a malignancy-suspect lesion according to the BI-RADS methodology to justify a biopsy of the lesion, which is considered the gold standard in the diagnosis of suspected breast lesions. Breast imaging methods only raise suspicion of malignancy. Histopathological confirmation or exclusion of malignancy should be pursued (6). Women with breast lesions without suspicion of malignancy in any of these three methods methods in the electronic records were not subjected to histological examination of the lesion; therefore, they were not included in the study to compare the diagnostic accuracy of these imaging methods.

Although MRI can more accurately detect malignant breast lesions, we stress the fact that breast biopsy might be indicated in patients with equivocal findings in one of the three methods, who are anxious or have difficulties with regular follow-up. These reasons may have led to a breast biopsy in cases where only ultrasonography indicated that the lesion was suspicious for malignancy, generating false-positive results and reducing the specificity of this method in our study. As mentioned in our study, of the 13 false-positive results in the ultrasonography examination, 10 patients presented small nodules, making it difficult to adequately characterize them since little interobserver agreement has been reported in the ultrasonography characterization of small lesions, according to the BI-RADS methodology (7). Additionally, after reviewing the data, we observed that at least five of the false-positive results of ultrasonography were suspicious for malignancy in at least one of the other two methods, MRI or mammography, which might also have led the attending physician to indicate the biopsy.

Regarding the specificity of MRI, we emphasize in this study that this is a controversial issue, with variable results in different studies published in the literature. Thus, although in this study, it was not categorically stated that breast density was a limiting factor for the specificity of the method, it was shown that of the eight false-positive MRI results, six patients had this type of breast composition, suggesting an association. Such an association was justified in some studies by the superposition of morphological and kinetic characteristics between normal breast tissue, benign and malignant lesions, as well as by the high rate of proliferation alterations found in dense breasts (8,9). Therefore, although more recent studies have shown that sequences using the diffusion technique improve the sensitivity of MRI and that it is currently a methodology routinely used in the service where electronic medical records were archived, it cannot be stated that diffusion was used in all cases, as some of these cases were archived at least 10 years before the study was carried out and were not accessed at the time of image acquisition.
